# Exploring the Optimal Chemotherapy Strategy for Locoregionally Advanced Children and Adolescent Nasopharyngeal Carcinoma Based on Pretreatment Epstein-Barr Virus DNA Level in the Era of Intensity Modulated Radiotherapy

**DOI:** 10.3389/fonc.2020.600429

**Published:** 2021-01-07

**Authors:** Ziyi Zeng, Chen Chen, Lanlan Guo, Cheng Zhang, Lei Chen, Chuanping Yuan, Lixia Lu

**Affiliations:** ^1^ State Key Laboratory of Oncology in South China, Collaborative Innovation Center for Cancer Medicine, Guangdong Key Laboratory of Nasopharyngeal Carcinoma Diagnosis and Therapy, Sun Yat-sen University Cancer Center, Guangzhou, China; ^2^ Department of Radiation Oncology, Sun Yat-sen University Cancer Center, Guangzhou, China; ^3^ Department of Radiology, Sun Yat-sen University Cancer Center, Guangzhou, China; ^4^ Department of Oncology, Xinyu People’s Hospital, Xinyu, China

**Keywords:** nasopharyngeal carcinoma, children and adolescents, induction chemotherapy, concurrent chemotherapy, intensity, courses

## Abstract

**Background:**

The present study aimed to explore the optimal chemotherapy strategy for locoregionally advanced children and adolescent nasopharyngeal carcinoma (LcaNPC), based on the level of pretreatment plasma Epstein-Barr virus DNA (pEBV-DNA) in the era of intensity modulated radiation therapy (IMRT).

**Methods:**

This real-world, retrospective study consecutively reviewed locoregionally advanced nasopharyngeal carcinoma patients younger than 22 years old from 2006 to 2016 in the Sun Yat-sen University Cancer Center. The Kaplan–Meier method with the log-rank test and the Cox regression model were used to investigate the survival outcomes of different chemotherapy intensities and pEBV-DNA. Treatment-related toxicity was also evaluated using the chi-squared test or Fisher’s exact test.

**Results:**

A total of 179 patients were enrolled, including 86 patients in the high-risk group (pEBV-DNA ≥7,500 copies/ml) and 93 patients in the low-risk group (pEBV-DNA <7,500 copies/ml). Among all patients, those receiving low intensity induction chemotherapy (IC courses = 2) had a better 5-year overall survival (OS) than those receiving no IC (P = 0.025) and high intensity IC (IC courses >2) (P = 0.044). In the high-risk group, receipt of low intensity IC showed significant 5-year OS (P = 0.032), progression-free survival (PFS) (P = 0.027), and 5-year distant metastasis-free survival (DMFS) (P = 0.008) benefits compared with not receiving IC. Multivariate analyses identified that not receiving IC was a risk factor compared with low intensity IC for OS (hazard ratio (HR) = 10.933, P = 0.038) among all patients. Moreover, in the high-risk group, not receiving IC was a risk factor for 5-year OS (HR = 10.878, P = 0.038), 5-year PFS (HR = 5.705, P = 0.041), and 5-year DMFS (HR = 10.290, P = 0.040) compared to low intensity IC. There were no differences in survival for patients treated with or without concurrent chemotherapy.

**Conclusion:**

Two courses of platinum-based IC might be the optimal induction chemotherapy intensity to reduce risk of death, progression, and distant metastasis in patients with high pEBV-DNA levels.

## Introduction

Children and adolescent nasopharyngeal carcinoma (CaNPC) is a rare malignant tumor that accounts for 1–2% of all nasopharyngeal carcinoma (NPC) in endemic areas (1). Similar to adult NPC, the most common pathological type of CaNPC is also WHO Type II and III (undifferentiated) (1). However, in contrast to adult NPC, CaNPC is more likely to be locally advanced, but has a better prognosis. In terms of overall stage, the 5-year overall survival rate of CaNPC is 83–89.2% compared to 62–83.6% in adult patients with NPC (2, 3). Given that a standard treatment has not been established, the treatment strategy for CaNPC mainly follows the treatment guidelines for adult NPC.

Induction chemotherapy (IC) combined with concurrent chemoradiotherapy (CCRT) has been the standard treatment for locoregionally advanced NPC in adults based on two large-scale prospective studies (4, 5). Several retrospective studies have also confirmed the efficacy and safety of IC plus CCRT in CaNPC (6–12). However, these studies do not show whether patients with CaNPC could actually benefit from IC. Recently, a matched cohort analysis by Li et al., which compared IC plus CCRT with CCRT alone in CaNPC, failed to demonstrate that adding IC before CCRT could provide a significant additional survival benefit (13). Meanwhile, the radiotherapy techniques used in that study varied, including two-dimensional conventional radiotherapy(2D-CRT), three-dimensional conformal radiotherapy (3D-CRT), and intensity modulated radiation therapy (IMRT), which might have had an impact on treatment outcomes. Moreover, there was no stratified analysis based on the courses of IC in that study. Therefore, the role of IC in CaNPC remains controversial.

Epstein-Barr virus (EBV) infection, which is known to be related to adult NPC, also plays an important role in prognosis of CaNPC (1). Patients with CaNPC with high pretreatment plasma EBV DNA (pEBV-DNA) (≥7,500 copies/ml) had poor survival, which indicated that more intensive treatment might be needed for these patients. Therefore, the purpose of the present study was to explore the optimal chemotherapy strategy for CaNPC in terms of chemotherapy courses and the level of pEBV-DNA in the era of IMRT.

## Patients and Methods

### Population

Consecutive records of patients with non-distant metastatic nasopharyngeal carcinoma (age ≤21 years old) were reviewed from January 2006 to December 2016 in the Sun Yat-sen Cancer Center. The records were collected independently from the case management system in our institution by two oncologists. Patients were pooled according to the following inclusion criteria: (1) Newly histologically diagnosed nasopharyngeal carcinoma; (2) locoregional advanced stage (III and IVa, according to the AJCC 8th edition stage system); (3) who received radical IMRT; and (4) with known pretreatment pEBV-DNA concentrations. Non-platinum based concurrent chemotherapy were excluded, which is considered a nonstandard therapy regime in our institution. This study was approved by the Institutional Review Board and the Research Ethics Committee of Sun Yat-sen University Cancer Center. Written informed consent to participate in this study was provided by the participants’ legal guardians or next of kin.

### Treatment

All patients received radical IMRT at our institution, the details of which were described previously (3, 13). The types of chemotherapy comprised IC alone, IC with CC, and CC alone. Several multidrug combined regimens were used for IC, including the TP regimen (docetaxel, 75 mg/m2 on day 1 and cisplatin, 80 mg/m2 on day 1), the PF regimen (cisplatin, 80 mg/m2 on day 1 and 5-fluorouracil, 800–1,000 mg/m^2^ for 96 h of continuous intravenous infusion), the TPF regimen (docetaxel, 75 mg/m2 on day 1 and cisplatin, 75 mg/m2 on day 1 with 5-fluorouracil, 750 mg/m^2^ for 96 h of continuous intravenous infusion), and the GP regimen (gemcitabine, 1 g/m2 on day 1 and 8 and cisplatin, 80 mg/m2 on day 1). All IC regimes (including TP, TPF, PF, and GP) were administered every 3 weeks for two to four cycles. The CC regimen was platinum based, and comprised cisplatin 80–100 mg/m2 every three weeks for two to three cycles or 30–40 mg/m2 every week for five to seven cycles during radiotherapy.

### Evaluation and Data Processing

All patients were restaged independently according to the 8th edition of the International Union Against Cancer/AJCC tumor-node-metastasis (TNM) staging system by two oncologists. Treatment-related toxicities were assessed according to the Common Terminology Criteria for Adverse Events version 4.0 (CTCAE 4.0). After treatment, all patients were followed up every 3 months during the first 2 years, every 6 months during the next 3 years, and then annually.

The patients’ pEBV-DNA concentrations were measured using quantitative real-time PCR (qPCR) before therapy (14, 15). According to their pEBV-DNA level, the patients were divided into a high-risk group (≥7,500 copies/ml) and a low risk group (<7,500 copies/ml) based on a previous study in our institution, which demonstrated that pEBV-DNA at more than 7,500 copies/mL was an independent unfavorable prognostic factor for survival in CaNPC (16).

According to IC courses, patients were divided into three groups: The no IC group, the low intensity IC group (IC courses = 2), and the high intensity IC group (IC courses = 3 or 4).

### Endpoints and Statistical Analysis

Overall survival (OS) was calculated from the beginning of therapy to death from any cause. Progression-free survival (PFS) was calculated from the beginning of therapy to recurrence or distant metastasis or death from any cause, whichever came first, and distant metastasis-free survival (DMFS), which was calculated from the beginning of therapy to first distant metastasis.

Survival analyses were conducted using the Kaplan–Meier method with the log-rank test. A Cox proportional hazards model was used for multivariate analysis with calculation of the hazard ratio (HR) and 95% confidence intervals (CIs). A chi-squared test (or Fisher’s exact test) was used to compare acute adverse events and late toxicities during treatment between the different treatment groups. A two-side *P* value less than 0.05 was considered as statistically significant. All statistical analyses were performed using IBM SPSS Statistics version 25 (IBM Corp., Armonk, NY, USA).

## Results

### Demographics

A total of 335 patients were screened, and 179 patients were finally included in this study. The baseline characteristics of the patients are listed in **Table 1**. The median age was 18 years old (range, 6–21 years old). Among T1–3 stages, one patient had T1 stage disease, 14 patients had T2 stage disease, and 115 patients had T3 stage disease. The numbers of patients with N0, N1, N2, and N3 stage disease were 10, 39, 105, and 25, respectively. There were 86 patients in the high-risk group (pEBV-DNA ≥7,500 copies/ml) and 93 patients in the low-risk group (pEBV-DNA <7,500 copies/ml).

**Table 1:** Baseline characteristics of the patients.

	All patients(n, %)	Low risk group(pEBV-DNA < 7,500 copies/ml) (n, %)	High risk group(pEBV-DNA ≥ 7,500 copies/ml) (n, %)	*P*
Age				0.590
≤17 years	87 (48.6)	47 (50.5)	40 (46.5)	
>17 years	92 (51.4)	46 (49.5)	46 (53.5)	
Sex				0.103
Male	136 (76.0)	66 (71.0)	70 (81.4)	
Female	43 (24.0)	27 (29.0)	16 (18.6)	
Histopathology				1.000
WHO II	3 (1.8)	2 (2.2)	1 (1.2)	
WHO III	176 (98.3)	91 (97.8)	85 (98.8)	
T stage				0.135
T1–3	130 (72.6)	72 (77.4)	58 (67.4)	
T4	49 (27.4)	21 (22.6)	28 (32.6)	
N stage				0.041
N0–1	49 (27.4)	31 (33.3)	17 (19.8)	
N2–3	130 (72.6)	62 (66.7)	69 (80.2)	
Overall stage				0.010
III	109 (60.3)	65 (69.9)	44 (51.2)	
IVa	70 (61.5)	28 (30.1)	42 (48.8)	
RT combined chemotherapy				0.160
IC	23 (12.8)	16 (17.2)	7 (8.1)	
IC+C C	119 (66.5)	56 (60.2)	63 (73.3)	
CC	34 (19.0)	20 (21.5)	14 (16.3)	
RT alone	3 (1.7)	1 (1.1)	2 (2.3)	
IC regimes				0.680
PF	37 (25.8)	17 (23.3)	20 (28.6)	
TP	28 (19.6)	16 (21.9)	12 (17.1)	
TPF	70 (49.0)	37 (50.7)	33 (47.1)	
GP	8 (5.6)	3 (4.1)	5 (7.2)	
CC				0.138
Yes	153 (85.5)	76 (81.7)	77 (89.5)	
No	26 (14.5)	17 (18.3)	9 (10.5)	
I.C intensity				0.247
No IC	36 (20.1)	20 (21.5)	16 (18.6)	
Low intensity IC	69 (38.5)	40 (43.0)	29 (33.7)	
High intensity IC	74 (41.3)	33 (35.5)	41 (47.7)	
Dose				0.886
≤68Gy	78 (43.6)	41 (44.1)	37 (43.0)	
>68Gy	101 (56.4)	52 (55.9)	49 (57.0)	

pEBV-DNA, pretreatment plasma EBV DNA; IC, induction chemotherapy; CC, concurrent chemotherapy; RT, radiotherapy.

### Survival Analysis Among All Patients

The cut-off date of follow-up was July 1, 2019. The median follow-up time was 56.1 months (range, 3.3–141.1 months). Among all 179 patients, 11 patients died, and 18 patients suffered from distant metastasis. Only three patients experienced locoregional recurrence. Among the patients with distant metastasis, six patients had bone metastasis, two patients had lung metastasis, one patient had liver metastasis, and nine patients had multiple site metastasis. Among patients with recurrent disease, one patient had nasopharyngeal recurrence, one patient experienced regional lymph node relapse, and one patient experienced both. The 1-year, 3-year, and 5-year OS rates were 98.9, 94.7, and 92.6%, respectively. The 1-year, 3-year, 5-year PFS rates and DMFS rates were 93.3 and 93.9%, 89.8 and 90.9%, and 88.4 and 90.2%, respectively.

**Figure 1:**
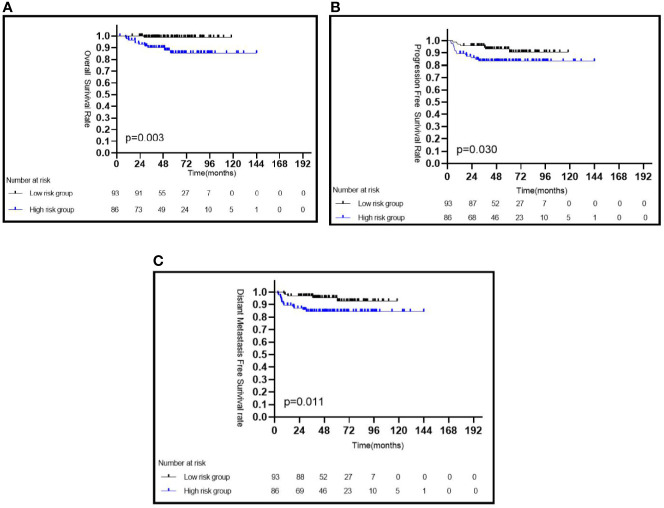
Kaplan–Meier survival curves of CaNPC in different risk groups based on pEBV-DNA **(A–C)**. pEBV-DNA, pretreatment Epstein-Barr virus DNA.

Patients with a low pEBV-DNA level had a significantly better 5-year OS than those with a high pEBV-DNA level (98.9 *vs.* 85.8%, *P* = 0.003) (**Figure 1A**), a better 5-year PFS (93.1 *vs.* 83.2%, *P* = 0.030) (**Figure 1B**), and a better 5-year DMFS (95.4 *vs.* 84.4%, *P* = 0.011) (**Figure 1C**).

**Figure 2:**
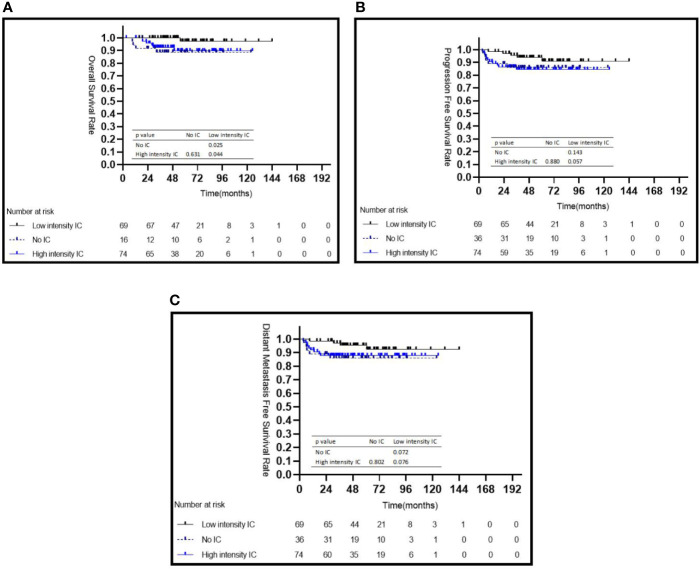
Kaplan–Meier survival curves for 179 patients with NPC stratified by intensity of induction chemotherapy **(A–C)**. IC, induction chemotherapy.

Among the three IC intensity groups, patients in the low IC intensity group had a better 5-year OS than those in the no IC group (98.0 *vs.* 88.6%, *P* = 0.025) or in the high intensity IC group (98.0 *vs.* 89.0%, *P* = 0.044) (**Figure 2A**), but showed no difference in the 5-year PFS and 5-year DMFS (all *P* > 0.05) (**Figures 2B, C**). No statistical differences were observed for the 5-year OS (*P* = 0.499), 5-year PFS (*P* = 0.395), and 5-year DMFS (*P* = 0.347) among patients treated with or without CC.

Univariate and multivariate analyses of 5-year OS, PFS, and DMFS among all patients are listed in **Table 2**. Through multivariate analyses, a high pEBV-DNA level was identified as an independent risk factor for 5-year OS (HR = 10.103; 95% CI: 1.250–81.680; *P* = 0.030) and 5-year DMFS (HR = 3.325; 95% CI: 1.009–10.470; *P* = 0.048). Compared with low intensity IC, no IC showed a risk for 5-year OS (no IC *vs.* low intensity IC: HR = 13.722, 95% CI: 1.422–132.426, *P* = 0.024), while high intensity IC failed to show a survival benefit (P = 0.133).

**Table 2:** Univariate and multivariate analysis for potential prognostic factors in clinical outcomes among all patients and patients in the high risk group.

Characters	5-yr OS			5-yr PFS			5-yr DMFS		
	*P1*	*P2*	HR (95% CI)	*P1*	*P2*	HR (95% CI)	*P1*	*P2*	HR (95% CI)
**All patients**									
Sex	0.796	0.358		0.940	0.525		0.965	0.481	
Male			1			1			1
Female			1.910 (0.481–7.585)			1.405 (0.492–4.012)			1.525 (0.472–4.930)
Age	0.781	0.657		0.586	0.569		0.751	0.644	
≤17 yr			1			1			1
>17 yr			0.739 (0.195–2.803)			0.766 (0.305–1.920)			0.785 (0.281–2.193)
T stage	0.910	0.522		0.452	0.520		0.293	0.461	
T1-3			1			1			1
T4			0.636 (0.159–2.542)			1.369 (0.526–3.563)			1.491 (0.515–4.319)
N stage	0.219	0.960		0.469	0.614		0.356	0.465	
N0-1			1			1			1
N2-3			2.9E5 (0.00–2.33E217)			1.345 (0.425–4.259)			1.644 (0.433–6.249)
pEBV-DNA	0.003	0.030		0.030	0.067		0.011	0.048	
<7500 copies/ml			1			1			1
≥7500 copies/ml			10.103 (1.250–81.680)			2.558 (0.937–6.985)			3.325 (1.009–10.470)
IC intensity	0.078	0.069		0.162	0.162		0.153	0.153	
Low intensity			1			1			1
No IC	0.025a	0.024a	13.722 (1.422–132.426)	0.143a	0.143a	3.203 (0.819–12.523)	0.072a	0.072a	3.887 (0.861–17.547)
High intensity	0.044b	0.130b	5.204 (0.613–44.149)	0.057b	0.057b	2.509 (0.814–8.306)	0.076b	0.076b	2.772 (0.731–10.508)
CC	0.574	0.747		0.921	0.480		0.600	0.989	
Yes			0.697(0.078–6.238)			0.628 (0.172–2.287)			0.989 (0.206–4.750)
No			1			1			1
**High risk group**									
Sex	0.234	0.339		0.733	0.507		0.630	0.340	
Male			1			1			1
Female			1.975 (0.489–7.994)			1.578 (0.411–6.058)			1.1.952 (0.494–7.706)
Age	0.961	0.679		0.899	0.836		0.884	0.994	
≤17 yr			1			1			1
>17 yr			0.748 (0.189–2.963)			0.888 (0.290–2.721)			0.995 (0.290–3.417)
T stage	0.136	0.491		0.534	0.821		0.150	0.901	
T1–3			1			1			1
T4			0.611 (0.150–2.482)			1.250 (0.384–4.073)			0.968 (0.271–3.454)
N stage	0.279	0.969		0.368	0.830		0.670	0.739	
N0–1			1			1			1
N2–3			3.02E5 (0.0–1.40E281)			1.055 (0.262–4.257)			2.335 (0.0.432–12.612)
IC intensity	0.118	0.118		0.095	0.095		0.039	0.039	
Low intensity			1			1			1
No IC	0.037a	0.037a	10.878 (1.135–104.273)	0.027a	0.027a	5.705 (1.073–30.340)	0.028a	0.028a	10.290 (1.115–94.968)
High intensity	0.137b	0.137b	3.758 (0.430–32.854)	0.179b	0.179b	2.821 (0.569–13.985)	0.072b	0.072b	5.584 (0.676–46.151)
CC	0.279	0.979		0.582	0.449		0.268	0.645	
Yes			1.32E5 (0.00–NA)			0.526 (0.100–2.771)			0.541 (0.095–3.071)
No			1			1			1

P_1_ is the p value for univariate analysis, P_2_ is the p value for multivariate analysis, HR, hazard ratio; CI, confidence interval; IC, induction chemotherapy; CC, concurrent chemotherapy; RT, radiotherapy; pEBV-DNA, plasma Epstein-Barr virus DNA; OS, overall survival; PFS, progression-free survival; DMFS, distant metastasis-free survival.

a is the comparison between the low IC intensity group and the no IC group, b is the comparison between the low IC intensity group and the high IC intensity group.,

### Survival Analysis Stratified by pEBV-DNA

**Figure 3:**
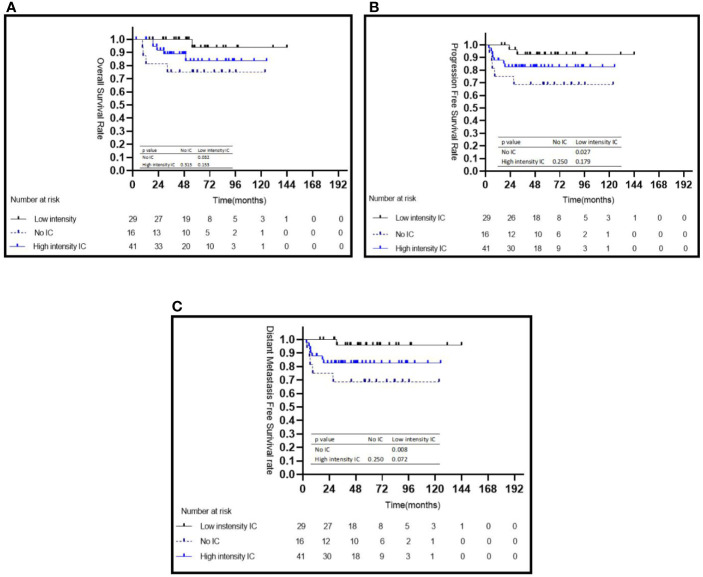
Kaplan–Meier survival curves for the subgroup of 86 patients with NPC in the high-risk group stratified by intensity of induction chemotherapy **(A–C)**. IC, induction chemotherapy.

The characteristics of the patients in two risk groups are listed in **Table 1**. In the high risk group, patients treated with low intensity IC achieved a significantly better 5-year OS (95.2 *vs.* 75.0%, *P* = 0.032) (**Figure 3A**), PFS (92.4 *vs.* 68.8%, *P* = 0.027) (**Figure 3B**), and DMFS (96.0 *vs.* 68.8%, *P* = 0.008) (**Figure 3C**) than those who did not receive IC; however, no statistically significant survival differences were observed when compared with the high intensity IC group (**Figures 3A–C**). No statistically significant differences were observed between the with or without CC groups in terms of 5-year OS (84.2 *vs.* 100%, *P* = 0.279), 5-year PFS (83.8 *v*s. 77.8%, *P* = 0.582), and 5-year DMFS (79.7 *vs.* 67.7%, *P* = 0.593) (**Table 2**). In multivariate analyses, not receiving IC was a risk factor for 5-year OS (HR = 10.878, 95% CI: 1.135–104.273, *P* = 0.038), 5-year PFS (HR = 5.705, 95% CI: 1.073–30.304, *P* = 0.041), and 5-year DMFS (HR = 10.290, 95% CI: 1.115–94.968, *P* = 0.040) compared with the risk of receiving low intensity IC. No other independent prognostic factors were identified in patients treated with or without CC (the details are shown in **Table 2**).

**Figure 4:**
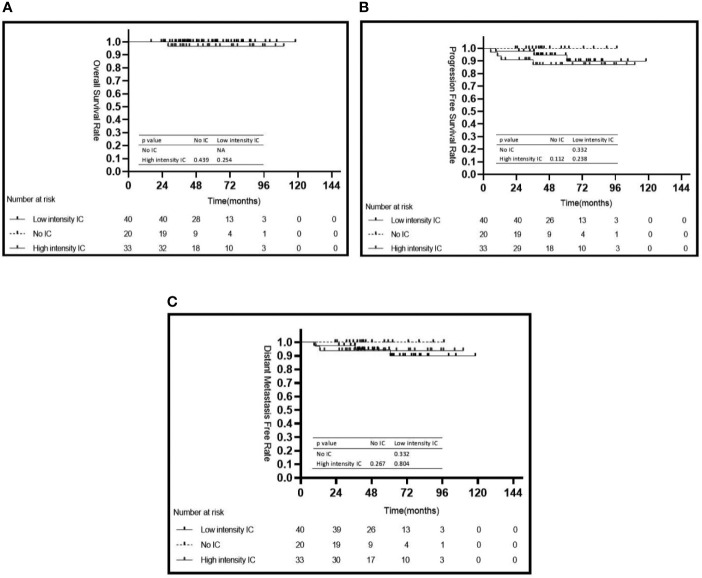
Kaplan–Meier’s survival curves for the subgroup of 93 patients with NPC in the low risk group stratified by intensity of induction chemotherapy **(A–C)**. IC, induction chemotherapy.

In the low risk group, there were no significant differences for 5-year OS, 5-year PFS, and 5-year DMFS in the comparison of the IC intensity groups or with/without CC groups (**Figure 4** and **Table 3**).

**Table 3:** Univariate and multivariate analysis for potential prognostic factors in clinical outcomes for patients in the low risk group.

Characters	5-yr OS			5-yr PFS			5-yr DMFS		
	*P1*	*P2*	HR (95% CI)	*P1*	*P2*	HR (95% CI)	*P1*	*P2*	HR (95% CI)
Sex	0.514	0.581		0.899	0.889		0.824	0.652	
Male			1			1			1
Female			0.023 (0.000–1.54E4)			0.884 (0.155–5.035)			0.574 (0.051–6.423)
Age	0.312	0.642		0.412	0.679		0.321	0.657	
≤17 yr			1			1			1
>17 yr			0.011 (0.000–1.93E6)			0.692 (0.121–3.946)			0.588 (0.056–6.145)
T stage	0.573	0.967		0.319	0.392		0.029	0.299	
T1–3 T4			1 0.471 (0.000–8.35E14)			1 2.230 (0.355–13.996)			1 3.144 (0.361–27.344)
N stage	0.468	0.653		0.296	0.340		0.618	0.509	
N0–1			1			1			1
N2–3			106.04 (0.000–7.29E10)			3.038 (0.309–29.823)			2.331 (0.189–28.681)
IC intensity	0.387	0.891		0.182	0.435		0.554	0.736	
Low intensity			1			1			1
No IC	NAa	0.882a	47.686 (0.000–7.84E23)	0.332a	0.978a	0.000 (0.000–NA)	0.332a	0.985a	0.000 (0.000–NA)
High intensity	0.254b	0.634b	261.50 (0.000–2.41E14)	0.238b	0.154b	3.739 (0.611–22.8)	0.804b	0.433b	2.483 (0.255–24.179)

P_1_ is the p value for univariate analysis, P_2_ is the p value for multivariate analysis, HR, hazard ratio; CI, confidence interval; IC, induction chemotherapy; CC, concurrent chemotherapy; RT, radiotherapy; pEBV-DNA, plasma Epstein-Barr virus DNA; OS, overall survival; PFS, progression-free survival; DMFS, distant metastasis-free survival.

a is the comparison between low IC intensity group and no IC group. b is the comparison between low IC intensity group and high IC intensity group.

### Toxicity Analysis

Acute adverse events were assessed in all 179 patients (**Table 4**). The most common hematological toxicity was leukocytopenia (n = 164, 91.6%), followed by neutropenia (n = 130, 72.6%). As the intensity of IC increased, the proportion of acute adverse events increased. The proportion of grade 3–4 neutropenia was significantly higher in the high intensity IC group (43.2%) than that in the low-intensity IC (24.6%) or no IC group (11.1%) (*P* = 0.001).

**Table 4:** Cumulative acute adverse events during therapy by maximum grade per patient during therapy.

Adverse event	No IC (n=36)						Low intensity IC (n=69)						High intensity IC (n=74)						*p* 1-4		*p* 3-4
(toxicity grade)	1	2	3	4	1–4	3–4	1	2	3	4	1–4	3–4	1	2	3	4	1–4	3–4			
	(%)	(%)	(%)	(%)	(%)	(%)	(%)	(%)	(%)	(%)	(%)	(%)	(%)	(%)	(%)	(%)	(%)	(%)			
Leucocytope- nia	12	11	8	0	31	8	11	28	20	2	61	22	13	41	18	0	72	18	0.046	0.467	
	(33.3)	(30.6)	(22.2)	(0.0)	(86.1)	(22.2)	(15.9)	(40.6)	(28.2)	(2.8)	(88.4)	(31.9)	(17.6)	(55.4)	(24.3)	(0.0)	(97.3)	(24.3)			
Neutropenia	4	6	4	0	14	4	14	21	12	5	52	17	12	20	22	10	69	32	0.000	0.001	
	(11.1)	(16.7)	(11.1)	(0.0)	(38.9)	(11.1)	(20.3)	(30.4)	(17.4)	(7.2)	(75.3)	(24.6)	(16.2)	(27.0)	(29.7)	(13.5)	(93.2)	(43.2)			
Anemia	21	4	1	0	26	1	36	15	4	0	55	4	49	17	2	0	68	2	0.021	0.610	
	(58.3)	(11.1)	(2.8)	(0.0)	(72.2)	(2.8)	(52.2)	(21.7)	(5.8)	(0.0)	(79.7)	(5.8)	(66.2)	(23.0)	(2.7)	(0.0)	(91.9)	(2.7)			
Thrombocyto- penia	5	3	0	0	8	0	7	10	0	0	17	0	5	9	2	0	16	2	0.907	0.356	
	(13.9)	(8.3)	(0.0)	(0.0)	(22.2)	(0)	(10.1)	(14.5)	(0.0)	(0.0)	(24.6)	(0)	(6.8)	(12.2)	(2.7)	(0.0)	(21.6)	(2.7)			
AST increase	1	0	0	0	1	0	18	0	0	0	18	0	18	0	1	0	19	1	0.010	1.000	
	(2.8)	(0.0)	(0.0)	(0.0)	(2.8)	(0)	(26.1)	(0.0)	(0.0)	(0.0)	(26.1)	(0)	(24.3)	(0.0)	(1.4)	(0.0)	(25.7)	(1.4)			
ALT increase	10	1	0	0	11	0	26	7	1	0	34	1	35	9	1	0	45	1	0.012	1.000	
	(27.8)	(2.8)	(0.0)	(0.0)	(30.6)	(0)	(37.7)	(10.1)	(1.4)	(0.0)	(49.3)	(1.4)	(47.3)	(12.2)	(1.4)	(0.0)	(60.8)	(1.4)			
BUN increase	5	1	0	0	6	0	10	0	0	0	10	0	16	0	1	0	17	1	0.228	1.000	
	(13.9)	(2.8)	(0.0)	(0.0)	(16.7)	(0)	(14.5)	(0.0)	(0.0)	(0.0)	(14.5)	(0)	(21.6)	(0.0)	(1.4)	(0.0)	(23.0)	(1.4)			
Mucositis	10	9	4	0	23	4	13	17	7	0	37	7	12	16	6	0	34	6	0.204	0.856	
	(27.8)	(25.0)	(11.1)	(0)	(63.9)	(25.0)	(18.8)	(24.6)	(10.1)	(0)	(53.6)	(10.1)	(16.2)	(21.6)	(8.1)	(0)	(45.9)	(8.1)			
Dermatitis	6	1	0	0	7	1	20	3	1	0	24	1	10	3	1	0	14	1	0.062	0.636	
	(16.7)	(2.8)	(0)	(0)	(19.4)	(2.8)	(29.0)	(4.3)	(1.4)	(0)	(34.8)	(1.4)	(13.5)	(4.1)	(1.4)	(0)	(18.9)	(1.4)			
Vomiting	13	5	0	0	18	0	34	6	1	0	41	1	47	6	1	0	54	1	0.046	0.636	
	(36.1)	(13.9)	(0)	(0)	(50.0)	(0)	(49.3)	(8.7)	(1.4)	(0)	(59.4)	(1.4)	(64.5)	(8.1)	(1.4)	(0)	(73.0)	(1.4)			

Late toxicities were assessed in 168 patients (late toxicities could not be assessed in the 11 patients who died) (**Table 5**). As a special age group, the late adverse toxicities of radiotherapy focused on children. Therefore, grade 2–4 late toxicities were counted in our study, which could have long term, even lifelong effects on quality of life of child patients. The most common late radiotherapy-related toxicity was grade 2–4 xerostomia (n = 58, 34.5%), followed by grade 2–4 hearing impairment (n = 38, 22.6%) and grade 2–4 neck fibrosis (n =36, 21.4%). Ten (6.0%) patients suffered from endocrine dysfunction, three in the No IC group, four in the low intensity group, and three in the high intensity group. The incidence of late toxicities was not significantly different among the three IC intensity groups.

**Table 5:** Radiotherapy-related grade 2–4 late toxicities.

Late toxicities	No IC (n = 32,%)	Low intensity IC (n = 68,%)		High intensity IC (n = 68,%)	*P*
(grade 2–4)					
Xerostomia	12 (37.5)	23 (33.8)	23 (33.8)		0.925
Hearing impairment	8 (25.0)	16 (23.5)	14 (20.6)		0.884
Neck fibrosis	7 (21.9)	14 (20.6)	15 (22.1)		0.976
Chronic otitis	4 (12.5)	9 (13.2)	10 (14.7)		0.947
Trismus	3 (9.3)	7 (10.2)	5 (7.4)		0.828
Eye damage	3 (9.3)	3 (4.4)	7 (10.3)		0.384
Endocrine dysfunction	3 (9.3)	4 (5.9)	3 (4.4)		0.640
Temporal lobe injury	3 (9.3)	5 (7.4)	1 (1.5)		0.127

## Discussion

As far as we know, the efficacy of IC has not been evaluated well in locoregionally advanced CaNPC in the IMRT era and a standard combined chemotherapy strategy has also not been established. In the present real-world study with a large number of consecutive patients, we found that low intensity IC (two courses of platinum-based IC) was the optimal intensity of IC to reduce the risk of death, progression, and distant metastasis in patients with high pretreatment pEBV-DNA levels; however, there was no optimal IC intensity for patients with low pEBV-DNA.

The patients in our study achieved a satisfactory 5-year OS rate (92.6%), which was higher compared with that reported in a previous study from the same institution (86.6% in the IC +CCRT group and 80.2% in the CCRT group) (13). In the study by Li et al., patients who received 2D-CRT were also included, but we only included patients with IMRT, who have a better survival rate compared with those receiving 2D-CRT. Moreover, our outcome was similar to that reported by Lu et al. (92.8%), who studied the 5-year OS rate of 34 children patients treated with IMRT.

IC followed by CCRT had been widely accepted as the standard treatment strategy for locoregionally advanced NPC in adults, based on two large-scale prospective, multicenter, randomized phase III clinical trials (3, 4). However, there has been no prospective study to confirm the efficacy of IC in terms of survival in CaNPC. In the past two decades, several small-scale prospective studies have been conducted in CaNPC, which focused on reducing the dose of radiotherapy by using induction chemotherapy to shrink the tumor to reduce radiotherapy-related toxicities (9, 12, 17). These studies showed that three or four cycles of platinum-based IC could achieve a 76–98% overall response rate; however, they did not present results on the impact of IC on survival (9, 12, 17, 18). CaNPC is a rare disease; therefore, most studies on the impact of IC on survival have been retrospective. A matched cohort analysis by Yang et al. demonstrated that adding IC before CCRT not only had no significant additional survival benefit, but also increased toxicities, such as grade 3 to 4 neutropenia (13). Another retrospective analysis by Zheng et al. found that additional IC was not an independent factor for survival in a Cox proportional hazards regression model (19). It is worth noting that the IC delivered in the above studies had no survival benefit in CaNPC as opposed to that in adults, which might be associated with the tolerance of patients with CaNPC. The toxicity, together with high-intensity chemotherapy, might offset the survival benefits. Unfortunately, none of the above studies analyzed the effect of IC intensity on survival. Therefore, in the present study, we divided the patients into a no IC group, a low intensity IC group (IC courses = 2), and a high intensity IC group (IC courses = 3 or 4). The results showed that patients in the low IC intensity group had a better 5-year OS than those in no IC and high intensity IC groups. In addition, higher IC intensities resulted in the more acute adverse events. These results suggested that two courses of IC might be the optimal IC intensity. Univariate analysis suggested that patients in the high intensity IC group had a worse 5-year OS rate compared with those in the low intensity IC group; however, multivariate analysis suggested that high intensity IC was not a risk factor compared with low intensity IC. We found that patients treated with high intensity IC had more advanced disease compare with patients treated with low intensity IC (IVa stage: 44.6 *vs.* 40.6%, N3: 20.3 *vs.* 14.5%), and patients with more advanced stage disease were more likely to receive more courses of IC.

Pretreatment pEBV-DNA is an important biomarker to predict survival and guide treatment for adults with NPC (20, 21). We demonstrated a similar role of pEBV-DNA in survival prediction in CaNPC, which agreed with the result of a previous study (16). An observational study by Shen et al. showed that the pretreatment pEBV-DNA load was an independent prognostic indicator for DMFS and OS in pediatric patients with NPC. In terms of overall survival, the cutoff value of pretreatment pEBV-DNA = 7,500 copies/ml was suggested for CaNPC in a retrospective analysis by Shen et al., which was also used in the present study (16). The difference in survival associated with pretreatment pEBV-DNA suggested that the treatment intensity of patients with different pretreatment pEBV-DNA levels could be individualized, which has been explored in adults with NPC (21). However, there has been no similar study in CaNPC focusing on the IC intensity and survival stratified by pEBV DNA. In the present study, patients with high pretreatment pEBV-DNA levels had better 5-year OS, PFS, and DMFS when treated with low intensive IC compared to those treated with no or high intensity IC. This indicated that two courses of IC might be sufficient for patients with CaNPC with a high pretreatment pEBV-DNA level, which might be related to CaNPC being more sensitive to platinum-based chemotherapy (18). Higher intensity IC (more than two courses) did not show survival benefits; however, it did result in more adverse events, which could be attribute to immune system damage (22, 23).

Concurrent cisplatin chemotherapy during radiotherapy has been the standard treatment for locoregional advanced NPC for twenty years, following the publication of the phase 3 randomized intergroup study 0099 (24). Recently, a prospective study showed that CaNPC treated with IC+CCRT achieved a good 5-year OS rate (89.2%) (12). However, in the present study, we failed to find that patients treated with CC achieved better survival. On the one hand, there were only 26/179 patients that did not accept CC, which was a small percentage compared with the patients treated with CC. On the other hand, patients treated with CC were more likely to have stage IVa disease compared with patients treated without CC (41.1 *vs.* 26.9%), and patients with stage IVa might have worse survival.

The limitations of this study were mainly related to its retrospective nature. First, this was a single-center retrospectively non-randomized study and potential confounding factors might bias the results. Consequently, we conducted multivariate analyses to weaken these confounding effects. Second, the IC regimens varied because they were extracted directly from electronic records rather than decided upon by the authors. Based on this consideration, the aim of this study did not include determining the best IC regimen. Moreover, an international randomized, phase II study showed that patients with CaNPC treated with the IC regimes TPF or PF had no difference in survival (11). Finally, no head-to-head comparison of survival outcomes was made among different chemotherapy intensities in this study. We believe that such comparisons should be conducted in a well-designed randomized controlled trial with robust methodological support and the potential ability for practice-changing.

## Conclusion

The two courses of platinum-based IC might be the optimal chemotherapy intensity to reduce risk of death, progression, and distant metastasis in patients with high pEBV-DNA levels. Higher intensity IC increased toxicity without any survival benefit.

## Data Availability Statement

The original contributions presented in the study are included in the article/supplementary material. Further inquiries can be directed to the corresponding author.

## Ethics Statement

The studies involving human participants were reviewed and approved by Institutional Review Board and the Research Ethics Committee of Sun Yat-sen University Cancer Center. Written informed consent to participate in this study was provided by the participants**’** legal guardian/next of kin.

## Author Contributions

LL, ZZ, and CC designed the study. ZZ, GL, ZC, and YH collected the data. All authors discussed the data. ZZ and CC drafted the manuscript. All authors contributed to the article and approved the submitted version.

## Funding

This study was funded by the Planned Science and Technology Project of Guangdong Province (grant no. 2016A020215085) and the 308 Clinical Research Funding of Sun Yat-sen University Cancer Center (grant no. 308-2015-011).

## Conflict of Interest

The authors declare that the research was conducted in the absence of any commercial or financial relationships that could be construed as a potential conflict of interest.

## Abbreviations

LcaNPC, locoregionally advanced children and adolescent nasopharyngeal carcinoma; CaNPC, children and adolescent nasopharyngeal carcinoma; NPC, nasopharyngeal carcinoma; pEBV-DNA, plasma Epstein-Barr virus DNA; IMRT, intensity modulated radiation therapy; 2D-CRT, two-dimensional conventional radiotherapy; IC, induction chemotherapy; CC, concurrent chemotherapy; CCRT, concurrent chemoradiotherapy.
